# Supplementary material for: Incorporation of Bayberry Tannin into a Locust Bean Gum/Carboxycellulose Nanocrystals/ZnO Coating: Properties and Its Application in Banana Preservation
Source: Polymers (Basel). 2023 Aug 10;15(16):3364. doi: 10.3390/polym15163364 (PMC10458572; doi:10.3390/polym15163364)
Supplement: Supplementary file 1 [file polymers-15-03364-s001.zip › polymers-2542404-supplementary.pdf]

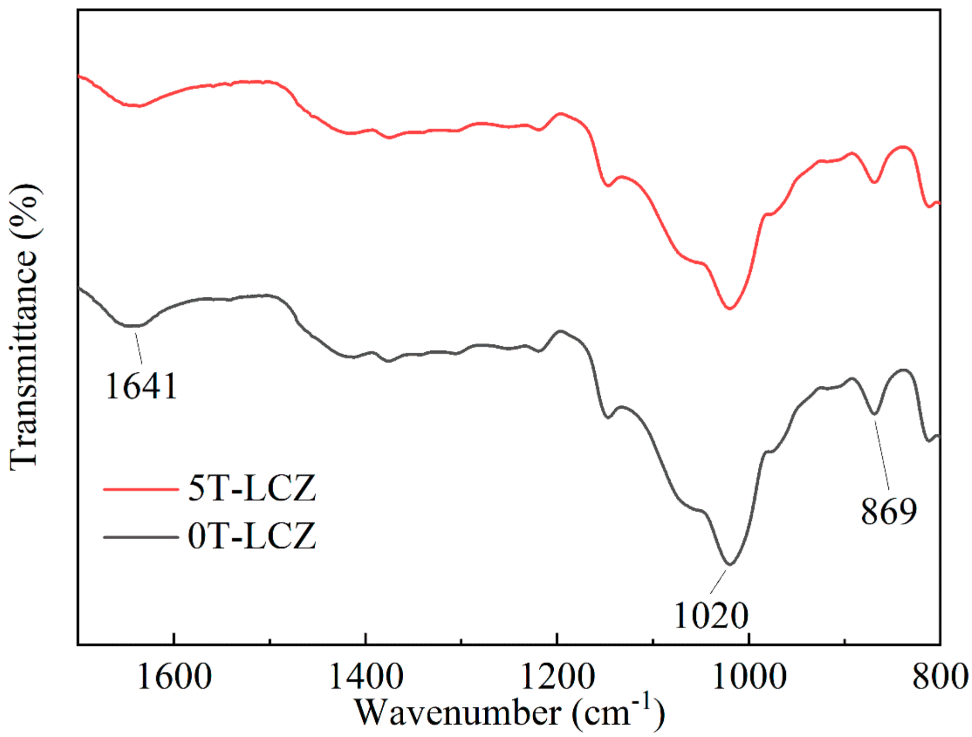

Figure S1. FTIR spectra at 1700-800 cm^{-1} for 0T-LCZ and 5T-LCZ.

As depicted in Fig. S1, the FTIR spectra of BT addition at 0% and 5% exhibit no significant difference because the characteristic bands of BT were covered by those of LCZ due to the smaller amount of BT. Moreover, they also provides an evidence that no chemical reaction occurred among the components.
